# Preparation of gamma poly-glutamic acid/hydroxyapatite/collagen composite as the 3D-printing scaffold for bone tissue engineering

**DOI:** 10.1186/s40824-022-00265-7

**Published:** 2022-05-31

**Authors:** Thu-Trang Nguyen, Chih-Chien Hu, Rajalakshmi Sakthivel, Sasza Chyntara Nabilla, Yu-Wen Huang, Jiashing Yu, Nai-Chen Cheng, Yi-Jie Kuo, Ren-Jei Chung

**Affiliations:** 1 Department of Chemical Engineering and Biotechnology, National Taipei University of Technology (Taipei Tech), Taipei, Taiwan; 2 Bone and Joint Research Center, Chang Gung Memorial Hospital, Linko, Taiwan; 3 Department of Orthopaedic Surgery, Chang Gung Memorial Hospital, Linko, Taiwan; 4 College of Medicine, Chang Gung University, Taoyuan, Taiwan; 5 Department of Materials, University of Oxford, Oxford, UK; 6 Department of Chemical Engineering, College of Engineering, National Taiwan University, Taipei, Taiwan; 7 Department of Surgery, National Taiwan University Hospital and College of Medicine, Taipei, Taiwan; 8 Department of Orthopedic Surgery, Wan Fang Hospital, Taipei Medical University, Taipei, Taiwan; 9 Department of Orthopedic Surgery, School of Medicine, College of Medicine, Taipei Medical University, Taipei, Taiwan

**Keywords:** Bone and osteochondral, 3D printing, Collagen, Polyglutamate acid, Hydroxyapatite

## Abstract

**Background:**

All types of movements involve the role of articular cartilage and bones. The presence of cartilage enables bones to move over one another smoothly. However, repetitive microtrauma and ischemia as well as genetic effects can cause an osteochondral lesion. Numerous treatment methods such as microfracture surgergy, autograft, and allograft, have been used, however, it possesses treatment challenges including prolonged recovery time after surgery and poses a financial burden on patients. Nowadays, various tissue engineering approaches have been developed to repair bone and osteochondral defects using biomaterial implants to induce the regeneration of stem cells.

**Methods:**

In this study, a collagen (Col)/γ-polyglutamate acid (PGA)/hydroxyapatite (HA) composite scaffold was fabricated using a 3D printing technique. A Col/γ-PGA/HA 2D membrane was also fabricated for comparison. The scaffolds (four layers) were designed with the size of 8 mm in diameter and 1.2 mm in thickness. The first layer was HA/γ-PGA and the second to fourth layers were Col/γ-PGA. In addition, a 2D membrane was constructed from hydroxyapatite/γ-PGA and collagen/γ-PGA with a ratio of 1:3. The biocompatibility property and degradation activity were investigated for both scaffold and membrane samples. Rat bone marrow mesenchymal stem cells (*r*BMSCs) and human adipose-derived stem cells (*h*ADSCs) were cultured on the samples and were tested *in-vitro* to evaluate cell attachment, proliferation, and differentiation. *In-vivo* experiments were performed in the rat and nude mice models.

**Results:**

*In-vitro* and *in-vivo* results show that the developed scaffold is of well biodegradation and biocompatible properties, and the Col-HA scaffold enhances the mechanical properties for osteochondrogenesis in both *in-vitro* and animal trials.

**Conclusions:**

The composite would be a great biomaterial application for bone and osteochondral regeneration.

## Introduction

Anatomical disruption of cartilage and underlying bone results in osteochondral defect (OCD) [1] due to repetitive microtrauma, ischemia, and the issue of genetic expression [2]. These defect may lead to disability and pain during every movement of the joint, and gradual deformation of bone [3]. Subchondral bone is heavily affected in such defects. This unique structure includes a large number of blood vessels supporting osteogenesis and maintains sufficient biomechanical support for the upper articular cartilage [4]. To date, microfracture, autologous implantation, autograft transfer, autograft transplantation, allograft transplantation, and bone tissue engineering have been the main surgical treatment methods for OCD. However, limited donor site morbidity, immune and inflammatory responses, as well as the risk of disease transmission are the major drawbacks for the use of autograft and allograft [5]. Tissue engineering is the ultimate method that is widely recognized for bone regeneration without depending on donor sources [6] owing to the use of transplantable scaffolds. An ideal scaffold for tissue engineering should possess excellent biocompatibility and induce osteogenic differentiation from multipotent mesenchymal stem cells (MSCs) [7] by providing the extracellular matrix and mimicking the tissue microenvironment. Evolving the conventional techniques, 3D printing has demonstrated its great potential for producing 3D-highly-porous functional scaffolds for biomedical applications. Extrusion printing is the most widely used 3D-bioprinting techniques with the direct-write printing feature [8] that facilitates the creation of new biocompatibility inks such as the vicious-polymer hydrogels.

Type I collagen (Col) is the major polymer in the connective tissues of hard bone, skin, and blood vessels, which plays an important role in bone reconstruction [9] via Arg-Gly-Asp sequences that are excellent specific bioligands for osteoblast adhesion with the interaction of α(2) β(1) integrins to stimulate the bone morphogenetic protein (BMP) [10]. The subchondral bone is a biphasic material. It comprises a hydroxyapatite (HA) crystal inorganic section for stiffness, and an organic section of principally type I collagen, proteoglycans, and glycosaminoglycans, and water, improving its elasticity and pliability [11] The bioactivity of HA is marked by osteoconductive and osteoinductive processes, which are involved in osseointegration support. The osteoinductive property of HA plays a guiding role in the formation of new bone on its surface down to the apertures of the implant body [12]. HA osteoconductivity improves the attachment, proliferation, growth, and phenotypic expression of osteoblast in a direct contact manner, hence a strong tissue-implant interface formation [13]. This property of HA stimulates tissue growth, which tolerates the bone neoformation, even in the non-bone-forming zone. In addition, the HA coating improves the initial mechanical stability post-implantation, which leads to a decrease in aseptic loosening. HA aids in the chemical bonding of the implanted tissue to native tissue with the absorbable protein on the surface of the implant. Protein attendance on the surface is promising for early healing at the tissue-implant interface [14]. Unfortunately, HA disperses homogeneity and aggregation within the polymer matrix resulting in nozzle clogging during printing and compromising the mechanical scaffold [5]. However, gamma-polyglutamic acid (γ-PGA) possesses an excellent water absorption ability and can absorb moisture up to 1400 – 5000 times its weight, facilitating its use as a wound-healing factor to achieve high levels of absorption of wound exudates [15]. Moreover, the L-isoform of γ-PGA has attracted increasing attention for biomedical applications because of its biodegradability and biocompatibility. Otherwise, this polymer includes the negative charge [16, 17] that possesses the largest affinity with the HA surface [18–20]. Therefore, γ-PGA is a great solution for dissolving HA to form an HA gel-like bioink structure.

Biodegradability and biocompatibility are the strengths of type I collagen for bone regeneration, but its most significant drawback is its poor low mechanical properties. Fortunately, oligo proanthocyanidins (OPCs) is a good candidate for cross-linking to improve the mechanical properties of collagen scaffolds via oligomers of catechin and epicatechin and their gallic acid esters. Nimni et al*.* [21] reported that OPCs enhances collagen synthesis and increase the speed of conversion of insoluble collagen from soluble collagen during development. Furthermore, OPCs possess various characteristics such as antioxidant, antiviral, antibacterial, anti-inflammatory, anticarcinogenic, vasodilatory actions, and anti-allergic properties. They can inhibit capillary hyperpermeability, platelet aggregation, and lipid peroxidation [22].

In this study, we aimed to fabricate the 4-layer scaffold with two printheads bio-printer to mimic the articular cartilage structure and focus on simulating the function of subchondral bone with high molecular interaction, with the first layer composed of HAp and γ-PGA equivalent subchondral bone layer, and the second to fourth layers comprised of type I collagen and γ-PGA similar to the superficial – middle –deep zone of articular cartilage. This scaffold was compared to collagen, collagen-γ-PGA scaffolds, and 2D membrane for biocompatibility and biodegradation in the in vitro experiments, then implanted into the rat and nude mice models. OPCs were used as biocompatible cross-linker for these scaffolds and membrane.

## Materials and methods

### Materials

Type I collagen from bovine skin was purchased from Devro Pty Limited (Bathurst, Australia), Hydroxyapatite was acquired from Acros organic (Thermofisher Scientific, USA), γ-Polyglutamate was obtained from Vedan company (Taiwan), Oligo Proanthocyanins (OPCs) extracted from grape seed that was purchased from Gino Biotechnology Ltd. (Taipei, Taiwan).

### Preparation of bioink for 3D printing

*Hydroxyapatite and Gamma- Polyglutamate*: First, 2 g of γ-PGA was completely dissolved in 10 mL of deionized water, then added 6.5 g HA was added to this solution and dissolved immediately until a slurry-gel-like structure was formed.

*Collagen and Gamma- Polyglutamate*: Acetic acid was used as a solvent for both Col and γ-PGA. To avoid the poly-ion complex aggregation of collagen and γ-PGA. First, 20 mg γ-PGA was dissolved in 5 mL deionized (DI) water by stirring to form a homogenous solution. Subsequently, 450 mg of Col powder was added, stirred vigorously to ensure that the powder was uniformly distributed in the γ-PGA solution. Subsequently, 5 mL of acetic acid was added to the solution. As the Col powder was already uniformly dispersed, the addition of acetic acid caused immediate dissolution of the Col powder, which prevented aggregation due to the γ-PGA/Col polyion complex. Thus, a homogeneous solution was obtained.

*Oligo Proanthocyanidin Solution:* 300 mg of OPC was dissolved in 30 ml of DI water to obtain a concentration of 10 mg/mL.

### Fabrication of 3D scaffolds and a 2-layer membrane of Col/γ-PGA/HA

**Fig. 1:**
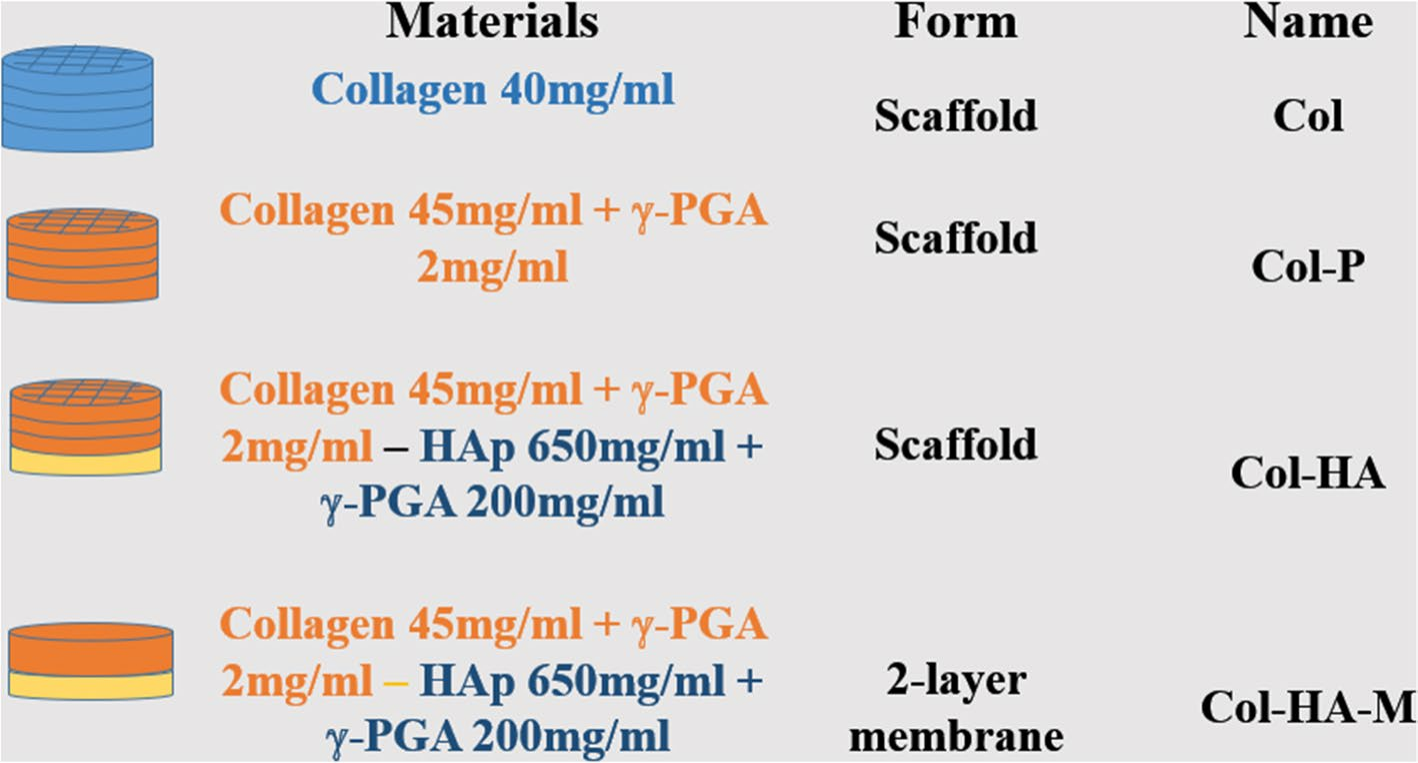
Fabrication of 3D scaffolds and 2-layer membrane with different compositions and concentration

Composite scaffolds of Col, γ-PGA, HA, and OPC were fabricated using a 3D printing machine (Cellink, USA). There were three combinations of scaffold composition: (I) Col with a concentration of 40 mg/mL [23] was then printed with a 30 mm length of 22G sterile needle (Cellink, USA), a pressure of 120 kPa, a speed of 4 mm/s. (II) Col with a concentration of 45 mg/ml was mixed with 2 mg/mL γ-PGA, then printed with a 30 mm length of 22G needle at a pressure of 90 kPa, perimeter speed of 4 mm/s, and infill speed of 3 mm/s. (III) The HA (650 mg/ml)/γ-PGA mixture (200 mg/ml) was printed as a first layer using a 24 mm length of 22G needle, the pressure of 90 kPa, perimeter and infill speed of 4 mm/s, and 3 mm/s, respectively, thereafter the collagen (45 mg/ml)/γ-PGA (2 mg/ml) was also printed as second to fourth layers using a 30 mm length of 22G needle, the pressure of 250 kPa, and speed of 3 mm/s to fabricate the scaffold of Col (45 mg/mL) combined with γ-PGA (2 mg/mL) and HA (650 mg/mL) together with γ-PGA (200 mg/mL). The scaffolds were soaked in OPC solution (10 mg/mL) for 4 h to obtain crosslinking. The scaffolds were freeze-dried for 24 h. Figure 1 illustrates the concentration of the fabricated 3D scaffolds and arrangement of each layer. In addition, the two-layers membrane of Col/γ-PGA/HA was also fabricated. The bottom layer consisted of a mixture of HA (650 mg/mL) and γ-PGA (200 mg/ml), then freeze-dried for 3 h. The collagen (45 mg/ml) and γ-PGA (200 mg/mL) were applied to the second layer. A combination of all mixtures was then soaked in the OPC solution (10 mg/mL) for 4 h and dried with a freeze dryer for 24 h.

### Characterization of the scaffolds and membrane

#### Fourier-transform infrared spectroscopy (FT-IR)

FTIR analysis was performed to detect possible changes in the structure of Col after oxidation with γ-PGA and HA. Data were obtained using an FTIR spectrometer (Perkin Elmer, USA) with wavenumbers ranging from 4000 to 500 cm^−1^.

#### X-Ray diffraction

The XRD measurement is using the range of 20 – 60° in 2 thetas (θ) with CuKα (λ = 0.15405 nm) radiation as the source at a rate of 2° /min and with a 1° glancing angle against the incident beam on the surface of the scaffold using the X-ray diffractometer (X’Pert3 Powder, PANalytical, Netherland) to detect the precipitation of apatite on the surface, which demonstrates the biological property of materials.

#### Field emission scanning electron microscope (FE-SEM)

An FE-SEM instrument (JEOL JSM-F100, USA) was used to observe the surface morphology of the scaffold and membrane. For simulated body fluid (SBF) immersion, apatite was deposited on the surface of the scaffolds and membrane. The samples were gold-sputtered before observation. The surfaces of samples were captured at different magnifications: 25x, 85x, and 100x.

#### Compressive mechanical property

The mechanical properties were measured using a Dynamic Mechanical analyzer Q800 (TA Instrument, USA). The scaffold specimens (Ø 8 × 2.5 mm) and membrane with dimensions of 8 × 8 × 2 mm (length × width × height) were loaded under ramp force from 0.2000 N/min to 18.0000 N of the clamp compression depending on the air Bearing Gas at 37℃. A uniaxial compressive force was applied to the hydrogel constructs until the point of failure. The compressive modulus was is then determined from the slope of the stress − strain curve. Each sample was measured at least in triplicates.

#### Rheological properties of hydrogels

The viscosity, storage modulus, and loss modulus of the Col, Col-P, HA-P hydrogels were measured with a Modular Compact Rheometer (MCR 302, Anton Paar, Austria) under the cone CP25-1 for 8.2 min at room temperature (25 ℃). The complex viscosity and modulus changes over the shearing frequency were recorded using the Start Rheoplus software (Version 3.62, Anton Paar GmbH, Graz, Austria).

### Degradation test, pore size, and porosity measurement

The degradation rates of the scaffold samples were studied by performing a degradation tests. The scaffolds were soaked in 50 mL of phosphate-buffered saline (PBS) at 37˚C on different days to evaluate the degradation activity. The weights of the scaffolds before and after soaking were used to calculate the percentage of degradation as follows:

1 $$Degradation\;rate\;\left(\%\right)= \frac{{W}_{w}-{W}_{0}}{{W}_{0}} x 100\%$$

where W_w_ is the weight after soaking and W_0_ is the original height.

The pore size and connector size were measured by ImageJ software. The porosity was calculated according to the formula:

2 $$Porosity\;\left(\%\right)= \frac{{W}_{wet}-{W}_{dry}}{{V}_{2}-{V}_{3}} x 100\%$$

where W_wet_ is the weight of the materials after soaking in ethanol for quick sorption, Wdry is the the weight of the freeze-dried materials, V2 is is the volume of the solvent after soaking the materials, and V3 is the volume of ethanol after the samples are taken out.

### Surface deposition of calcium phosphate apatite

The solution was prepared by dissolving NaCl, NaHCO_3_, KCl, K_2_HPO_4_, MgCl_2_.6H_2_O, CaCl_2_, and Na_2_SO_4_ in Tris–HCL buffer at pH 7.38 (37˚C). A bone-like apatite layer was allowed to nucleate and grow on the surface of the samples. After the completion of the 7, 14, and 21 days of incubation, the samples were taken out and freeze-dried. The apatite morphology was investigated with FE-SEM analysis, and elemental composition was analyzed using the XRD. Prior to FE-SEM, the samples were coated with a platinum layer.

### *r*BMSCs and* h*ADSCs for cell culture

*r*BMSCs were isolated from the bone marrow of Sprague Dawley (5 weeks old) rats. The bone marrow cells were flushed from SD rat femurs and tibias with Dulbecco’s Modified Eagle’s Medium (DMEM—Gibco, USA), supplemented with 10% FBS, 1% penicillin–streptomycin, 1% glutamine, and 1% non-essential amino acids. Cells were plated in a 75 cm^2^ flask and incubated at 37℃ with 5% CO_2_. After 4 h, the non-adherent cells and supernatant were removed. Thereafter, *r*BMSCs were purified and the medium was replaced every 72 h. Passages 3 – 6 of *r*BMSCs were used for all experiments.

*h*ADSC were obtained from the National Taiwan University Hospital [24] and cultured in Dulbecco’s Modified Eagle’s Medium- F12 (DMEM—Gibco, USA), supplemented with 10% FBS, 1% penicillin–streptomycin, 1% glutamine, and 1% non-essential amino acids. Cells were cultured at 37℃ under 5% CO2, and the medium was renewed every two days until confluence was reached.

### Cytotoxicity test

Cell counting kit – 8 includes WST – 8 (2-(2-methoxy-4-nitrophenyl)-3-(4-nitrophenyl)-5-(2,4-disulfophenyl)-2H-tetrazolium, monosodium salt). This compound produces a water-soluble formazan dye due to bioreduction in the presence of an electron carrier, 1-Methoxy PMS. Cellular dehydrogenases reduce WST-8 to an orange formazan product that is solubilized in culture medium. The amount of formazan produced was directly proportional to the number of living cells. Since the CCK-8 solution is very stable and has little cytotoxicity. Cell Counting Kit-8 allows sensitive colorimetric assays for the determination of the number of viable cells in the proliferation assay. Therefore, we used the CKK-8 kit to count the quality of *r*BMSC and *h*ADSC viability that is seeded with the membrane and scaffolds.

On the first day, the scaffolds and membrane were sterilized under UV light for 30 min and then placed in the 48-well plate with 200 μL medium in each well for their water absorption. The next day, aspirate all the old medium, then seeded 5 × 10^4^ *r*BMSC, *h*ADSC, and 1 ml medium to each well, the cell settled on the surface and inside the scaffolds and were incubated for 1, 3, 7 days. The medium was replaced with 100 μL and 10 μL of CKK-8 solution in each well, incubated at 37 ℃ and 5% CO2 for 4 h under protected light conditions. Then, transferred 100 μL of supernatant to a new 96-well plate to absorb at 450 nm wavelength.

### Fluorescence staining with 4’,6-diamidino-2-phenylindole dihydrochloride (DAPI)

DAPI is a fluorescent stain with photostability that labels DNA and allows easy visualization of the nucleus in interphase cells and chromosomes in mitotic cells. DAPI can associate with the minor groove of double-stranded DNA, with a preference for the adenine–thymine cluster via the permeable cell membrane. This was used to label the nuclear DNA of cell growth when seed *h*ADSC with scaffolds and membrane.

After three days incubation of 5 × 10^3^/mL cell, aspirate medium from each well, washed thrice with PBS solution, and the scaffolds and membrane were placed in a new 48-well plate. Next, add 500 μl 300 nM DAPI stain solution (Sigma) to each well under the light-protected condition, incubated for 15 min. After completion of the reaction, the scaffolds and membrane were washed thrice with PBS solution, carefully cut the scaffold with the long dimension to obtain the middle layer, transferred to a slide, and covered. Observed the fluorescence cell inside of composites under a fluorescence microscopy (Nikon Eclipse 50i).

### Gene expression

*r*BMSCs have capable of differentiation to specific tissue depending on their gene activities. In particular, seeding *r*BMSC with the direct biomaterials that can stimulate expression specific target genes. Here, we determined the expression levels of Collagen type I (F: TCCAAGGAAATGGCAACTCAGCTC; R: GAAACAGACGGGGCCAACC), Collagen type 2 (F: TCGCTGGTGCTGCTGACGCTGCTCG, R: CTGAGGGCCAGGAGGTCCTCTGG), Aggrecan (F: GGCCATGGTCCTTCTATGAC, R: TGTTGACGAACTCCTGTTCC), and BMP-2 (F: TGCACCAAGATGAACACAGC, R: GTGCCACGATCCAGTCATTC) of *r*BMSCs and compared them to those of the house of keeping gene GAPDH (F: GTGAAGCTCATTTCCTGGTATG, R: AACTGAGGGCCTCTCTCTTG) when it were seeded with the scaffolds and 2D membrane.

After 14 days incubation of *r*BMSCs at a density of 5 × 10^4^ *r*BMSC, harvested cell and extracted mRNA with RNaesy Mini Kit (Qiagen, Germany), measured RNA with 10 mM Tris–Cl, pH 7.5 for mRNA purification. cDNA was synthesized using RevertAid First Strand cDNA Synthesis Kit (Thermo Scientific, USA). Finally, RT-PCR was performed with Taqman™ Universal PCR Master Mix (Thermo Scientific, USA), PCR thermo cycle in the Applied Biosystems StepOnePlus ™ Real-Time PCR System includes step 1: 95℃ for 10 min, 95 ℃ for 15 s, 60℃ for 1 min (40 cycles) and step 2: 25℃ ∞. the results were analyzed by 2^−ΔΔCt^ method – where ΔΔCT = (C_T_,target–C_T_,_GAPDH_) experimental sample—(C_T_,_target_–C_T_, _GAPDH_) of control sample.

### Quantitative immunoassay

The 5 × 10^4^ of *h*ADSCs were seeded on the scaffolds and membrane at 2, 5, and 7 days. The medium was then switched to a serum-free medium for 24 h. The supernatant was tested with bone morphogenetic protein 2 (BMP-2) Quantikine enzyme-linked immunosorbent assay (ELISA) Kit (R&D System, USA) to evaluate BMP-2 concentration.

### In vivo experiment with nude mice and rat models and histological morphology

The biomaterials are assumed to direct the differentiation of BMSCs. To test this hypothesis, biomaterials should be implanted into the animal model and observed histologically using staining techniques. Sprague Dawley® (SD) rats (5-week-old) were used for in vivo experiments. The SD rats were acclimatized for at least one week before the experiment. The experiments were carried out at Chang Gung Memorial Hospital in accordance with guidelines for the care and use of animals. All experiments were approved by Affiliated Institutional Animal Care and Use Committee (IACUC) under Affidavit no. 2019102401. The diet was provided ad libitum with rat chow and continuously supplied with water. The histology of *r*BMSC and *h*ADSCs implanted with the Col, Col-P, Col-HA scaffolds, and Col-HA-M membrane was observed. The materials were seeded with 5 × 10^4^ *r*BMSC for 7 days. Following that, the scaffold and membrane were transplanted directly to the subcutaneous part of the back with four defects of the 15 Sprague Dawley® rats divided into three groups with different cages: Group I included one control rat, three material implanted rats, sacrificed after 1 week, group II was similar to the previous but sacrificed after 2 weeks, and group III included seven rats with one control rat and six rats carrying materials, and then sacrificed after 4 weeks. The scaffolds and membrane were harvested from the rat model after 1, 2, and 4 weeks and then sent to the Taipei Pathology Institute for histological observation via hematoxylin and eosin (H&E) staining.

The experiment with the nude mice model was similar, however, mice carried two scaffolds/ membranes. A total of 21 mice were investigated and divided into 3 groups, each group included 1 control mice and six material implanted mice, which were sacrificed after 1, 2, and 4 weeks, respectively. The samples were then subjected H&E staining to observe histology.

### Statistical analysis

In this study, the data are represented as the mean ± standard deviation. Student t-test two-tail was used for statistical analyses. *p* values < 0.05 were considered to be statistically significant. Origin 2019b software was used to evaluate the FTIR, XRD, mechanical properties, and rheological results. The pore size, fluorescence, and histological image were measured and edited by ImageJ.

## Results

### Characterization of the 2D membrane and 3D scaffolds

**Fig. 2:**
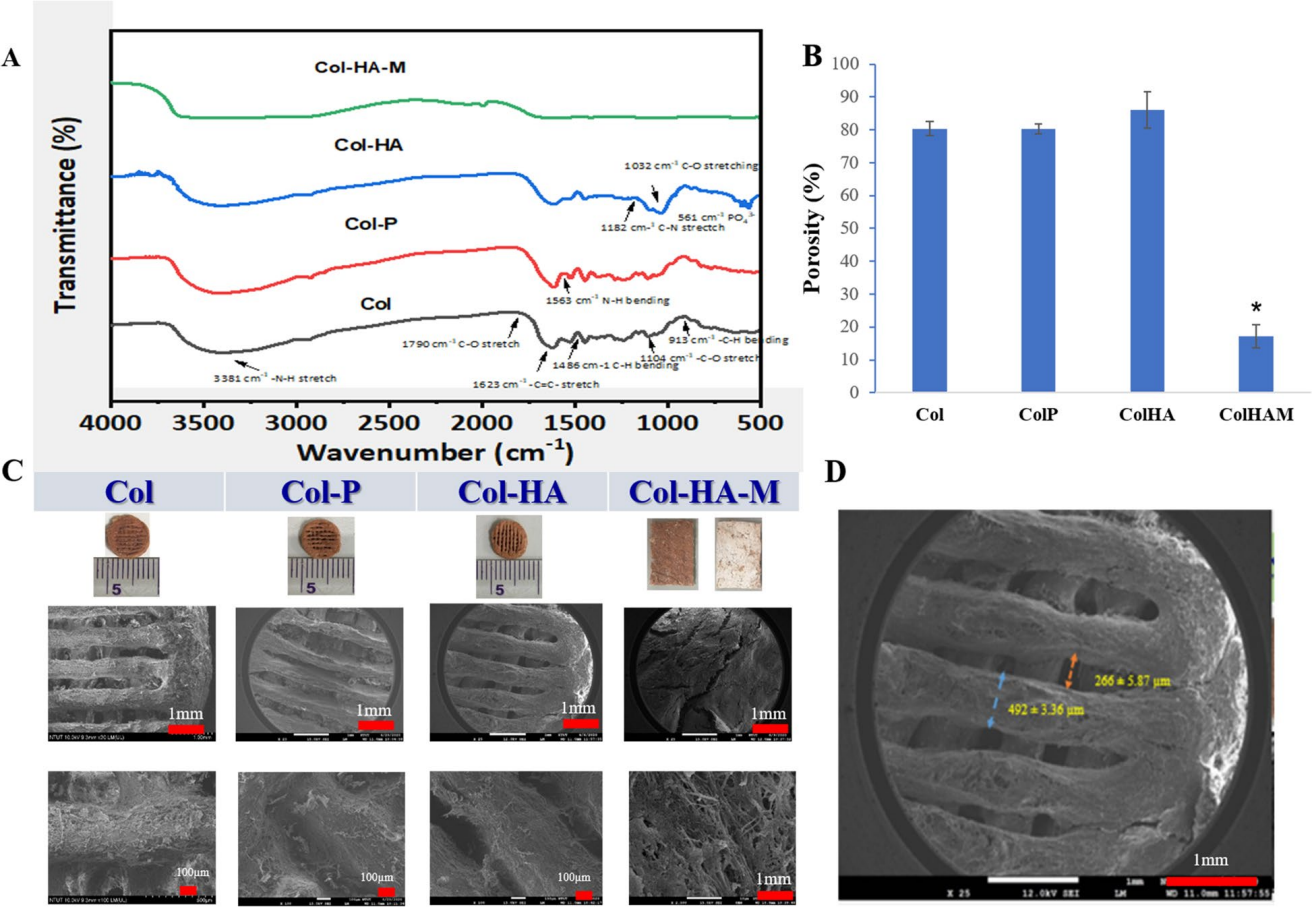
**(A)** FTIR spectra of collagen, collagen/γ-PGA, collagen/γ-PGA/hydroxyapatite 3D scaffolds, and the 2D membrane. **(B)** Porosity of materials. **(C)** Surface topography of scaffolds and the membrane **(D)** Pore size and connector size measurement (*n* = 5)

Figure 2A shows the FT-IR spectra of the three different scaffolds such as Col, Col-P, and Col-HA, and the membrane of Col-HA-M was immersed into the OPCs cross-linker. It can be seen that the type I collagen with OPCs shows the peaks at 3381 cm^−1^, 1790 cm^−1^, 1623 cm^−1^, 1486 cm^−1^, 1104 cm^−1^, 913 cm^−1^ assigned to the N–H stretching, C-O stretching, C = C stretching, C-H bending, C-O stretching, and -C-H bending, respectively. All the Col peaks were regenerated and also the new peak arose at 1563 cm^−1^ (N–H bending) signifying presence in the scaffold of Col-P. Besides the scaffold of Col-HA shows the Col peaks with one weak intense peak at 1182 cm^−1^ (C-N stretching) and two strong intense peaks at 1032 cm^−1^ (C-O stretching) and 561 cm^−1^ (PO_4_^3−^) which were attributed to the HA presence in the Col-HA scaffold. Finally, the Col-HA membrane shows weak intense peaks. This was due to the random arrangement of the Col-HA membrane.

Figure 2B-D presents the surface morphology of the scaffolds and membrane. In this study, the suitable pore size of our scaffolds was approximately 266 ± 5.87 µm (Fig. 2D), and the width of the connectors was 492 ± 3.36 µm (all of the scaffolds used the same needle size).

**Fig. 3:**
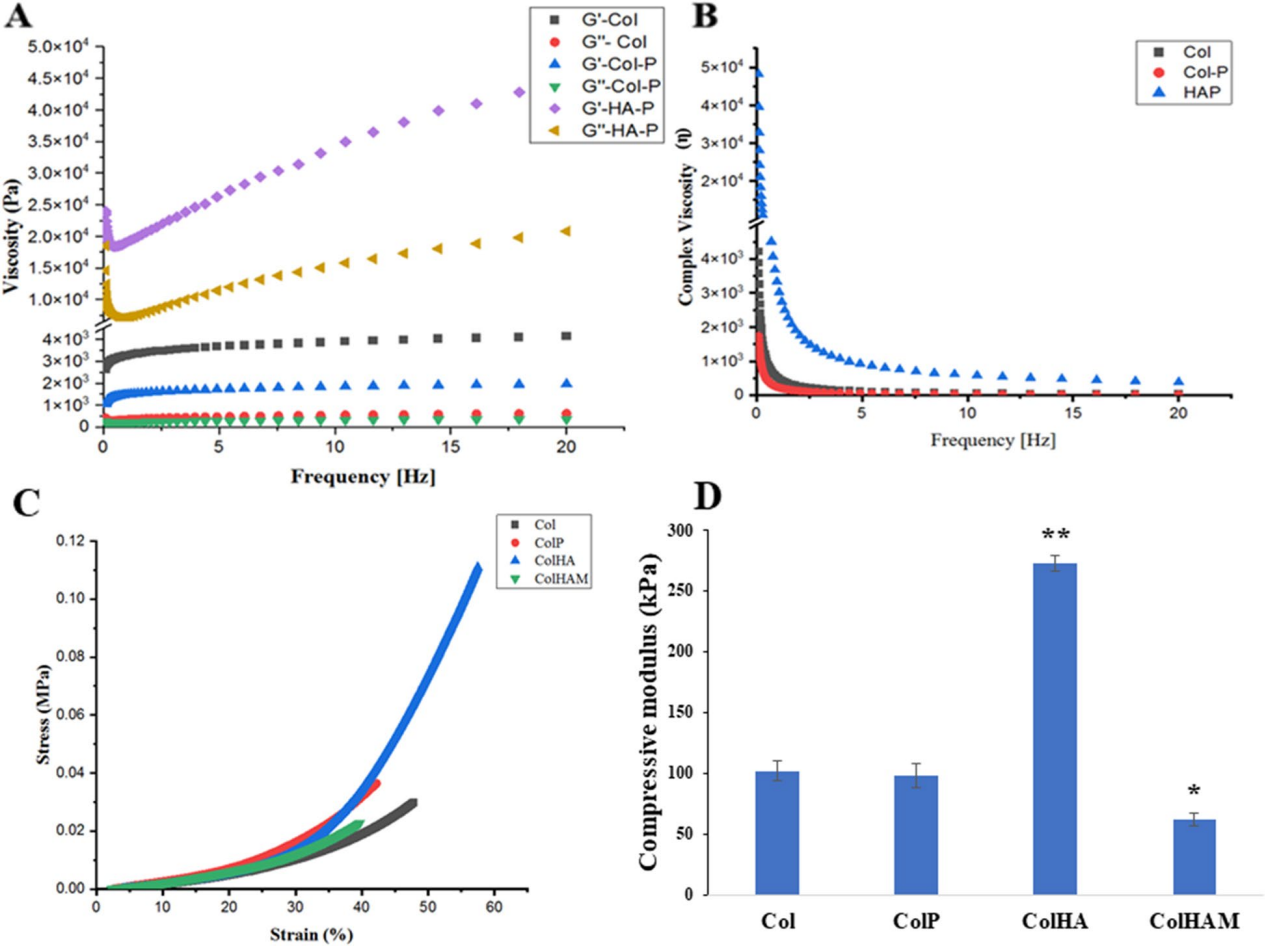
Rheological behavior of collagen (Col), Col-P, hydroxyapatite (HA)-P hydrogel. **A** Storage modulus (G’) and loss modulus (G’’). **B** Complex viscosity (η). Mechanical property of Col-HA membrane. **C** Compression testing stress–strain curve of scaffolds and membrane. **D** Compressive young’s modulus from the slope of the stress–strain curve (*n* = 3, * *p* < 0.05, ** *p* < 0.01)

The rheological properties of the hydrogels were evaluated, and the frequency was set in the range of 0 to 20 Hz for storage modulus and loss modulus testing (Fig. 3A-B). The minimum storage modulus of collagen hydrogel was approximately 270 Pa, whereas that of the Col – γPGA hydrogel was approximately 187 Pa. A possible reason is due to poly-electrolyte interaction between Col [25] and γPGA which reduce the viscosity of each other. For the HA – γPGA hydrogel, the significantly higher minimum storage modulus value at around 18.4 kPa because of the much higher concentration of γPGA, which is consistent with higher pneumatic pressure of HA-P hydrogel in 3D printing.

Figure 3C-D indicated the compression mechanical properties of the scaffolds and membrane that were conducted at 37℃ to mimic the bio-environment. The failure point of the 3D scaffolds was much higher than that of the membrane. The threshold point of the membrane was approximately 22.7 kPa while the Col and Col-P scaffolds were failed at around 49.3 and 36.46 kPa, respectively. The highest compressive stress was applied to the Col-HA scaffold is at about 111.09 kPa (Fig. 3C). Similarly, the compressive modulus of the specimens was reliable, and the compressive Young’s modulus of the Col-HA scaffold possesses the highest at 272 kPa. It was increased by three times compared to the Col and Col-P groups (102 and 97.8 kPa) and almost five times related to the Col-HA-M membrane (at 62.1 kPa). Overall, these results suggest that the Col-HA 3D scaffold has pretty good mechanical properties for bone regeneration and we used these materials for the following experiments.

### Biocompatibility of the scaffolds and 2D membrane

**Fig. 4:**
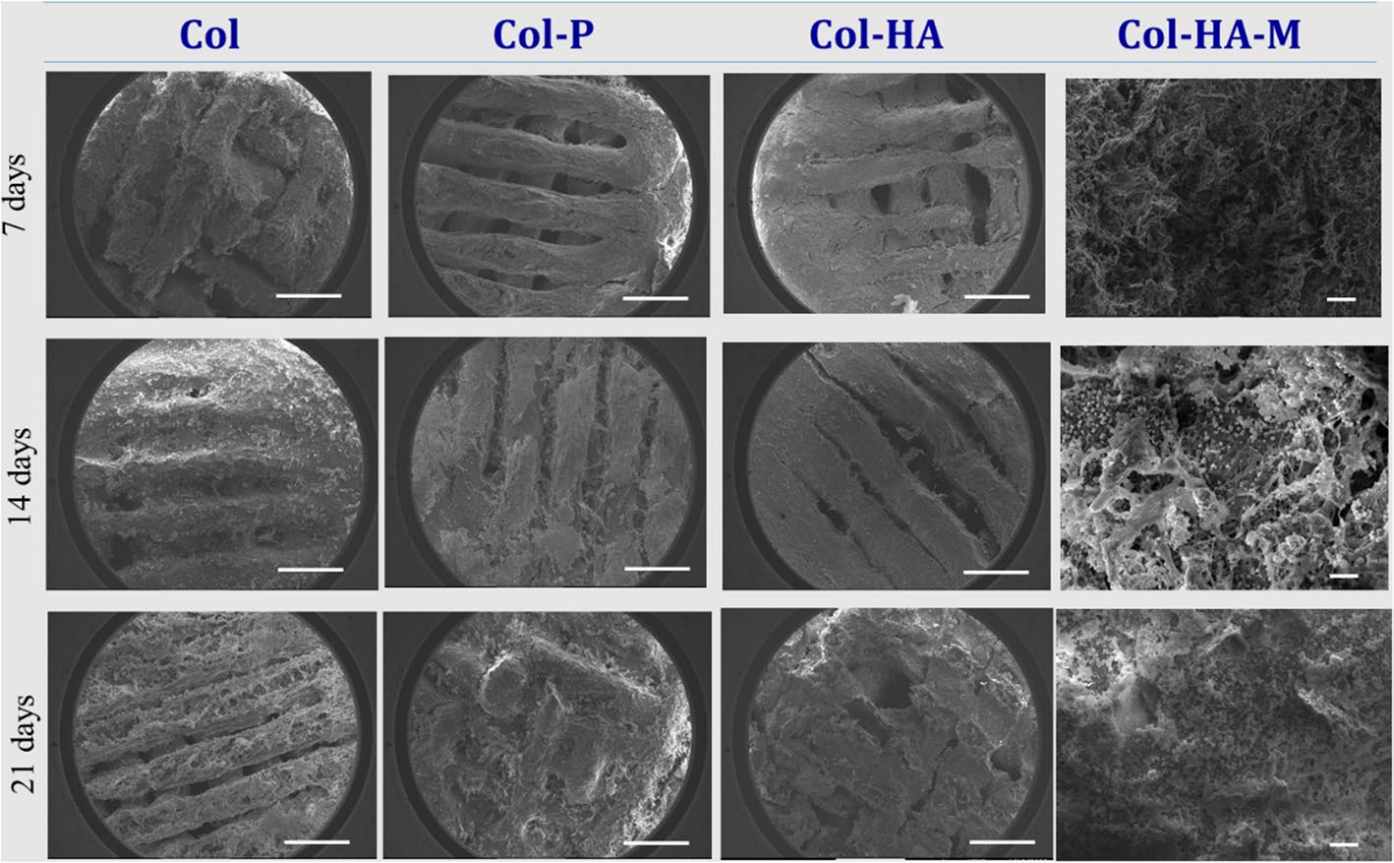
Apatite layers formation on the surface of 3D scaffolds (Col, Col-P, and Col-HA) and 2D membrane (Col-HA-M) after immersion in the simulated body fluid for 7, 14, and 21 days (Scale bar of 1 mm and 100 µm for the scaffold and membrane, respectively)

**Fig. 5:**
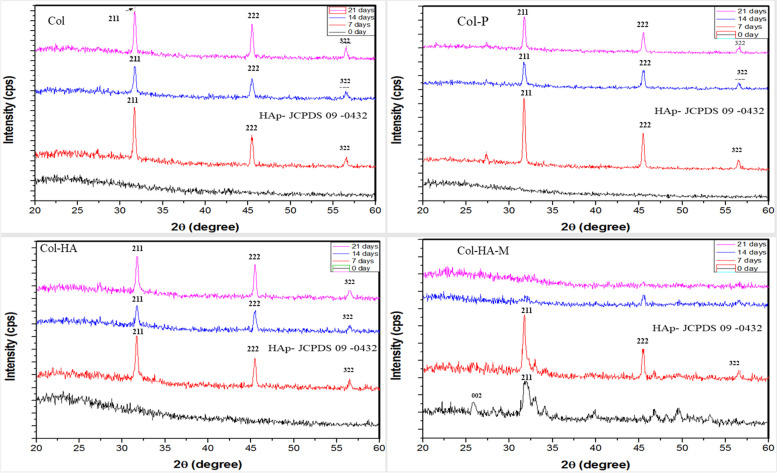
XRD patterns of the 3D scaffolds (Col, Col-P, and Col-HA) and 2D membrane (Col-HA-M) with peaks of appetite crystal structure as a function of SBF immersion time on 7, 14, and 21 days

Biomineralization of scaffolds in a SBF solution has become one of the most useful critical surface techniques for predicting the in vivo bone bioactivity of a material [26] by the deposition of apatite minerals, consequently increasing biocompatibility. Figure 4 shows the surfaces of Col, Col-P, and Col-HA scaffolds and Col-HA-M membrane. On day 7, the apatite formation in the Col-P and Col-HA scaffolds was higher than that of the Col surface due to the similar structure of Col-γPGA. The increase in the soaking time was attributed to more apatite precipitation on the surface. After 21 days, the empty region of the scaffolds was filled with apatite. This indicated that the scaffolds showed good biocompatibility. In terms of biocompatibility, all samples are suitable to be used for biomedical applications owing to their high apatite absorption. An XRD pattern was obtained for further confirmation (Fig. 5). According to JCPDS card No. 09–0432, it is found that the main diffraction peaks at 31.8°, 45.5°, and 56.5° 2θ can be indexed to the (211), (222), and (322) [27–29] planes matched the standard HA diffraction peaks. This indicated that the surface scaffolds were composed of apatite deposition.

### Biodegradability of composites

**Fig. 6:**
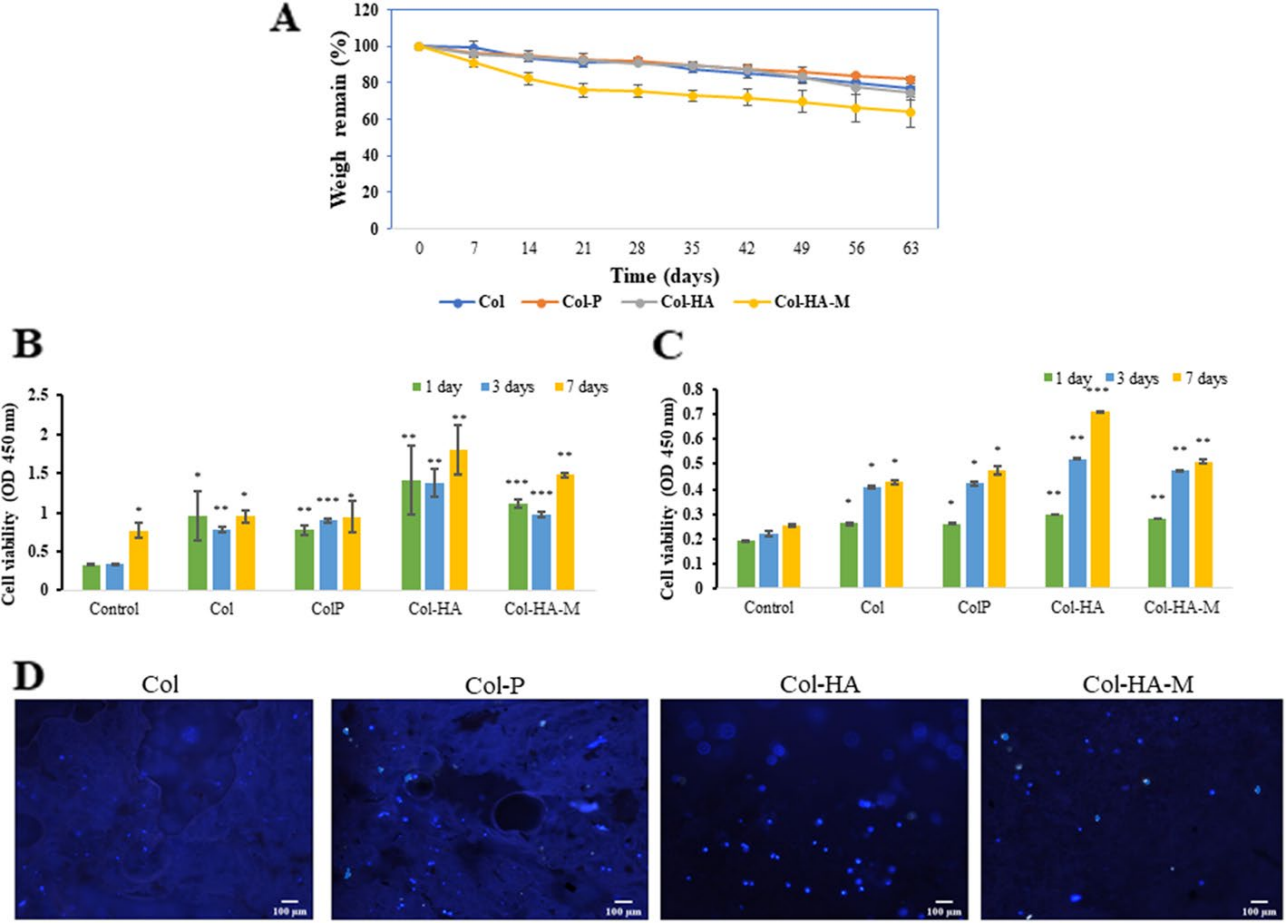
**A** Degradation rates of 3D scaffolds and 2D membrane as a function of time (*n* = 3). **B** Cell viability of *r*BMSCs and (**C**) *h*ADSCs on the Col, Col-P, and Col-HA scaffolds and Col-HA-M membrane. **D** Staining with 4’,6-diamidino-2-phenylindole dihydrochloride (DAPI) after seeding *h*ADSCs with materials for seven days (10X), the light blue dot indicates the nucleic (*n* = 5, * *p* < 0.05, ** *p* < 0.01, *** *p* < 0.001)

Figure 6A shows the degradation behavior of the scaffolds as a function of time until equilibrium was reached. The weight gradually decreased when the samples were immersed in PBS. The degradation rates of Col, Col-P, and Col-HA scaffolds were 92.0 ± 1.5%, 92.2 ± 0.9%, 90.77 ± 0.4%, respectively, with Col-HA reaching the highest rate of 76.6 ± 2.9% compared with the other scaffolds (76.6 ± 2.9% for Col and 81.9 ± 1.1%. for Col-P). The scaffolds were broken after 9 weeks of immersion. The degradation rate of the 2D Col-HA-M membrane was lower compared to 3D scaffolds. After 3 weeks, the degradation rate was 75.9 ± 3.6%. The equilibrium degradation rate is reached up to 36.06 ± 3.13%.

### Cell viability and cell proliferation

The viability of *r*BMSCs cultured in 3D scaffolds and 2D membrane was evaluated using the CKK-8 kit. The numbers of living cells were expressed as OD values. The number of living cells increased when seeded with the composition of both 3D scaffolds and 2D membrane (Fig. 6B), indicating higher cell proliferation. Col-HA scaffold exhibited an OD value of 1.80 ± 0.32 compared to other scaffolds such as 0.95 ± 0.08 and 0.94 ± 0.20 for Col and Col-P, respectively. According to Zhang et al*.* 2020 [30], the Col/hyaluronic acid gel used for cartilage generation showed an OD value of 1.1 after seven days of observation. In addition, Du et al*.* (2019) [6] reported that a mesoporous bioactive glass (MBG)/polycaprolactone (PCL) scaffold used for human bone marrow stem cells (*h*BMSCs) proliferation study demonstrated an OD value of 1.5. In this study, the Col-HA scaffolds presented higher OD values which were proven to be the microenvironment for the cells proliferation. Analogous results were obtained when *h*ADSCs seeded with our materials (Fig. 6C). The OD values of the Col-HA scaffold were the highest (0.7126 ± 0.0025) compared to those of other scaffolds (~ 0.15–0.51). Figure 6D demonstrates the DAPI staining results of the *h*ADSCs cultured on the Col, Col-P, Col-HA scaffolds as well as the Col-HA-M membrane. The density of nuclei and cytoskeleton development were higher developed on the Col-HA scaffold.

### Gene expression analysis and quantitative ELISA assay

**Fig. 7:**
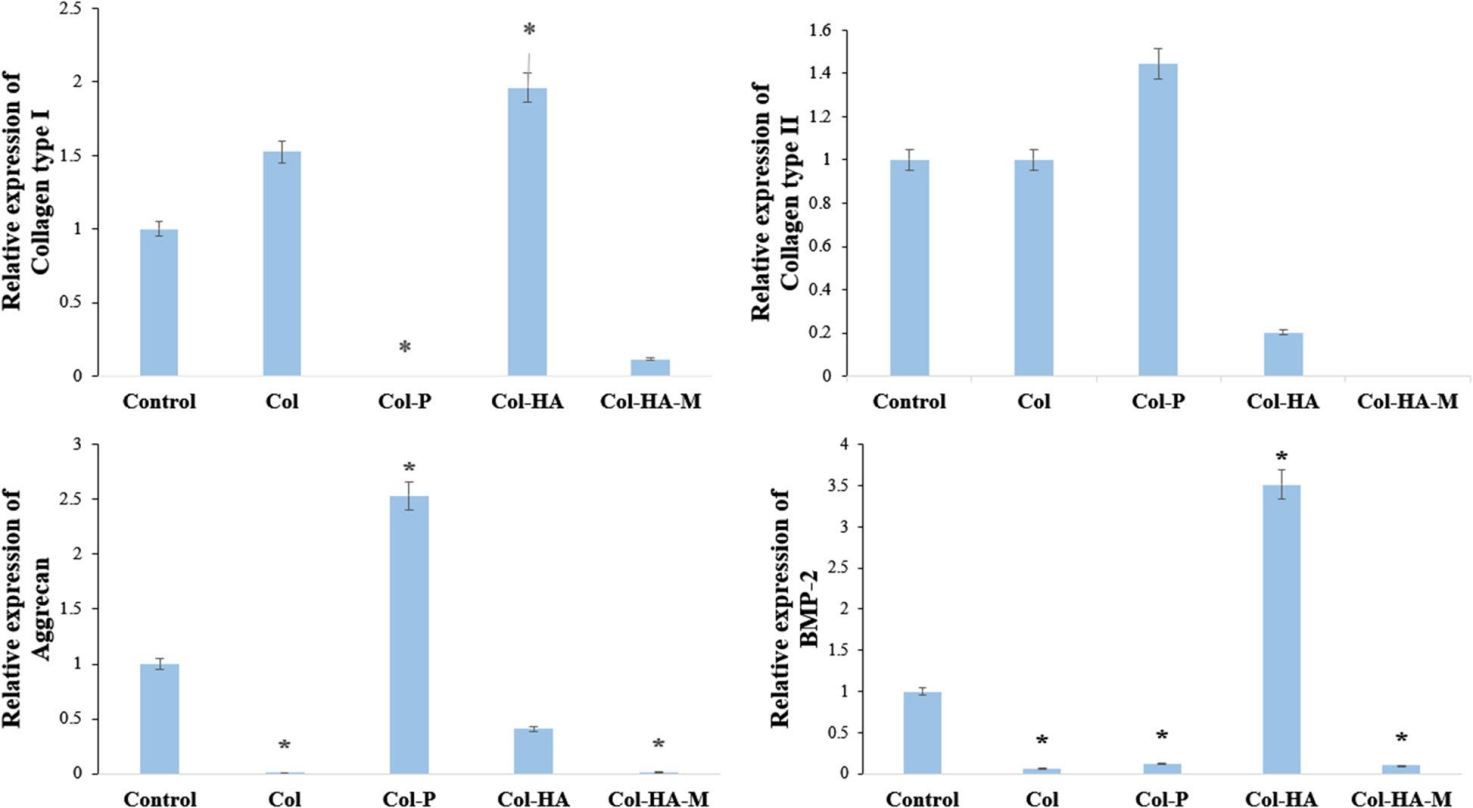
Gene expression for the differentiation of *r*BMSCs including Col I, Col II, Aggrecan, and BMP-2 after seeding on the scaffolds and membrane for 14 days. The genes of interest compared to those of the control-GAPDH (*n* = 3, * *p* < 0.05)

Expression of genes encoding type I and type II collagens, aggrecan, and BMP-2 was examined. As shown in Fig. 7, the majority of these genes are upregulated to different degrees in the *r*BMSCs cells cultured in 3D Col-based scaffolds, compared with those cultured on the 2D membrane, following culture for 14 days. The expression levels of type I collagen and BMP-2 in the Col-HA scaffold were upregulated and directed toward osteogenesis. Type II collagen and aggrecan were markedly upregulated in the Col-P scaffold which corresponded to chondrogenic for *r*BMCSs. Therefore, scaffold with HA composition promote osteogenic differentiation, and γ-PGA tends to induce chondrogenesis.

**Fig. 8:**
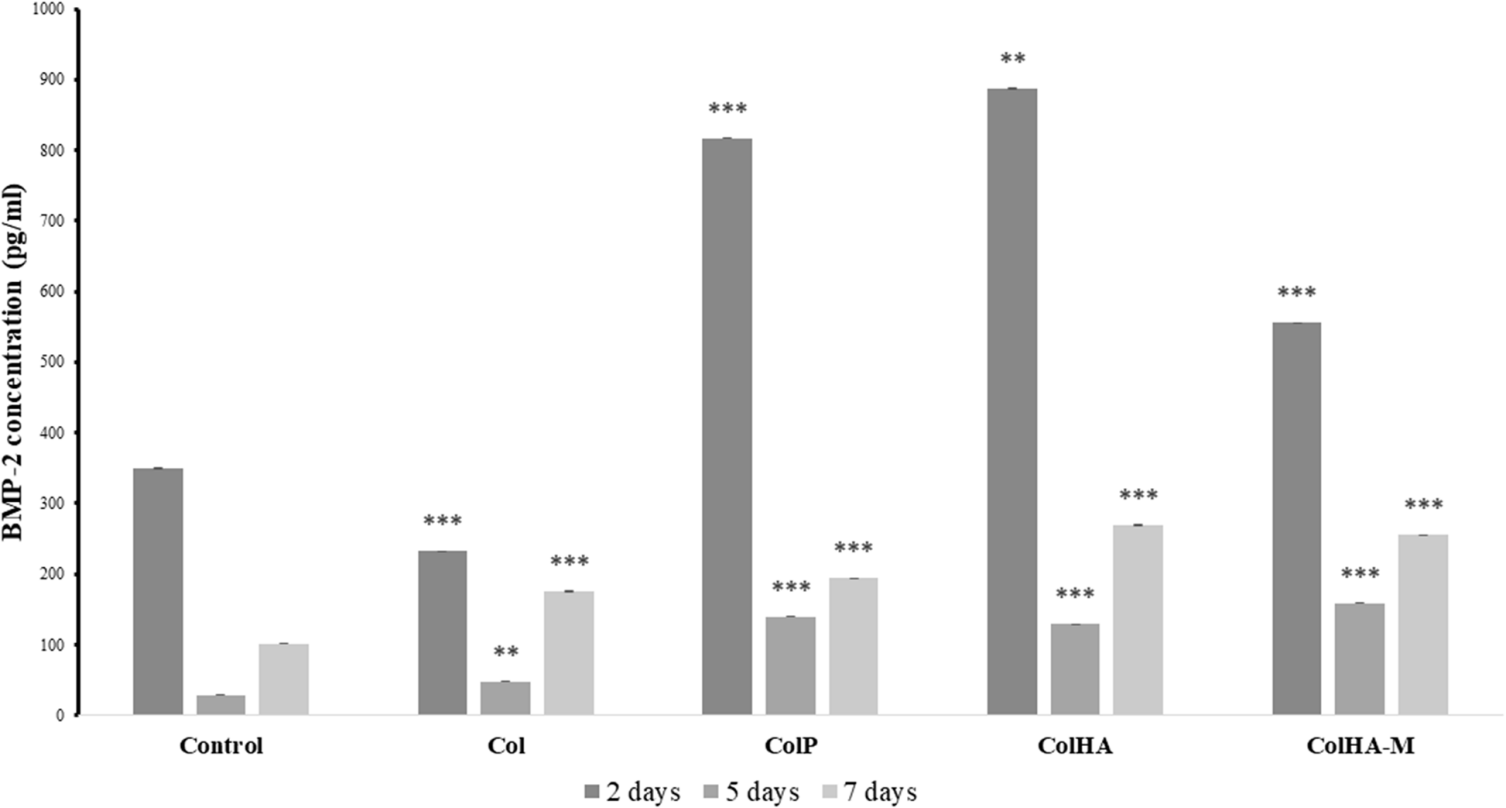
BMP-2 concentration of *h*ADSCs cultured on the 3D scaffolds and 2D membrane for 2, 5, and 7 days (*n* = 3, * *p* < 0.05, ** *p* < 0.01, *** *p* < 0.001).

Figure 8 demonstrates the amount of BMP-2 in the different groups. The Col-HA scaffold exhibited immense initial in the expression of the BMP-2 concentration after two days of culture. As the time progressed, the expression of BMP-2 significantly decreased on day 5 and then gradually increased on day 7 (Fig. 8). Compared with those in the corresponding 2D membrane, BMP-2 expression level in the 3D scaffolds were higher as follows the order of 817.17 ± 7.48 pg/ml, 556.25 ± 7.48 pg/ml, and 350.60 ± 7.477 pg/ml for Col, Col-P, and Col-HA-M, respectively. In general, all 3D scaffolds and membrane possessed good biocompatibility and biodegradability; however, the Col-HA scaffold indicates the most suitable properties for *in-vitro* bone regeneration in this study.

### In vivo biological evaluation

**Fig. 9:**
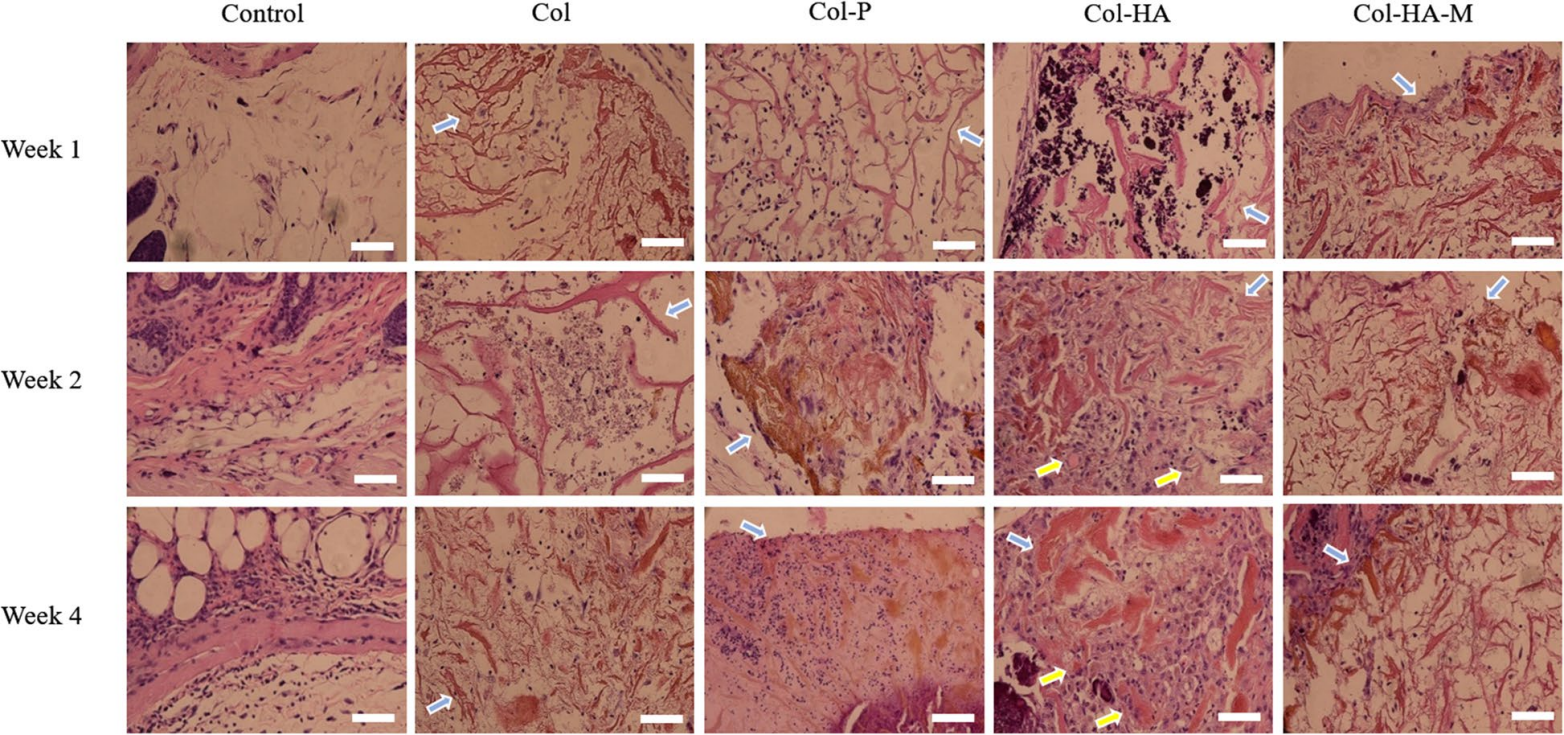
H&E staining of the collagen-based 3D scaffolds and 2D membrane implanted in the nude mice model after weeks 1, 2, and 4 (blue arrows indicate the scaffolding areas, yellow arrows indicate blood vessel development, and the scale bar size is 100 µm)

**Fig. 10:**
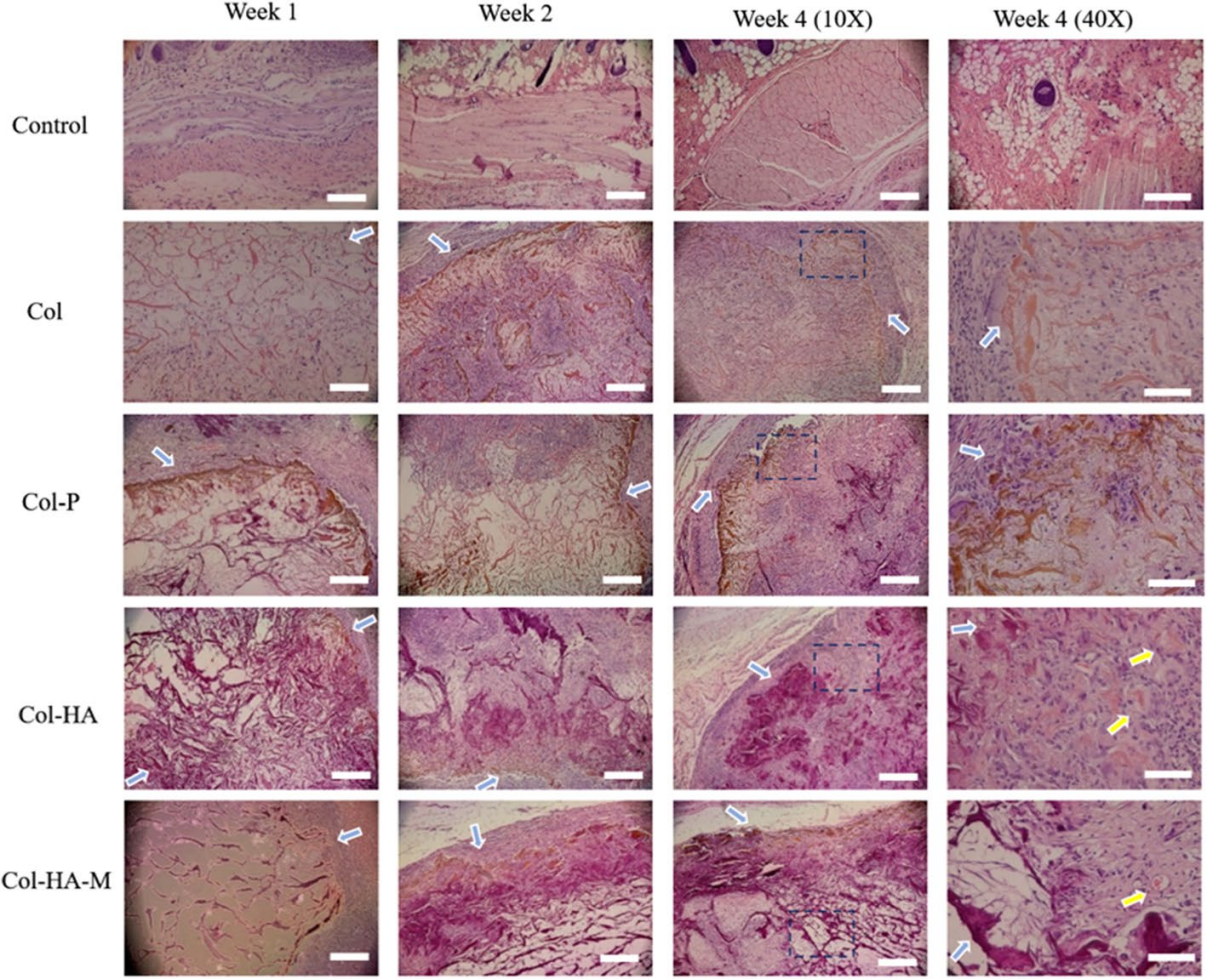
H&E staining of the collagen-based 3D scaffolds and 2D membrane implanted in the rat model after weeks 1, 2, and 4 (blue arrows indicate the scaffolding areas, yellow arrows indicate blood vessel development, and the scale bar size is 100 µm for 10X and 20 µm for 40X magnification)

The histology of the scaffolds and membrane which were implanted subcutaneously in the nude mice and rat models are presented in Fig. 9 and Fig. 10, respectively. The color of both 3D scaffolds and 2D membrane did not change (brown color); therefore, the barrier of seeded cells and native cells from the animal model could be recognized (blue arrow). After 4 weeks of implantation, the Col-HA scaffold was filled with cells (purple color indicates the nuclei) and ECM (pink color). The cell density expanded on the Col-HA scaffold was the highest compared to other scaffold compositions and even new blood vessel information (the yellow arrow), which exhibits the osteogenic character of subchondral bone because the osteocytes require vascular supply. However, the Col with γ-PGA showed more cell viability compared to the pure Col scaffold. In Col-HA-M, the cells appear in the opposite way with the cell core appearing on the surface of the membrane and the ECM inside it.

## Discussion

This study intended to develop a multilayer scaffold that serves for the OCD. The osteochondral bone consists of the subchondral bone plate, while the trabecular bone contains water and ECM. The ECM is composed of an organic matrix and inorganic HA which influence the elasticity and material stiffness of the bone, respectively [31]. One of the most vital roles of the subchondral is highly vascularized to facilitate the enrollment of progenitor cells. So, it has extraordinary inherent potential for spontaneous remodeling and regeneration. Moreover, the subchondral bone is a complex interface between bone and cartilage, which is a key challenge for OCD repair and regeneration. As such, in the current research, we aimed to develop a multilayer 3D printing scaffold that possessed improved biocompatibility and mechanical, osteogenic and chondrogenic properties.

From the bone tissue engineering point of view, high porosity of 3D printing scaffolds not only provides space for cell settlement and growth but also ensures the transport of nutrients and metabolites [7]. Generally, high porosities (> 80%) are evaluated as optimal for new bone tissue regeneration (Fig. 2B), and macroporosity with pore sizes in the range of 100 – 300 μm is beneficial for waste removal and nutrient supplementation [32]. Because the size of osteoblasts is on the order of 10—50 μm, therefore, a larger pore is preferred for regenerating mineralized bone after implantation [33]. In addition, small pores favor hypoxic conditions and induce osteochondral formation before osteogenesis occurs, whereas, larger pore scaffolds rapidly become well-vascularized leading to direct osteogenesis [34]. Moreover, the rheological properties of the bio-ink gel are a decisive factor for the porosities of the scaffold. Based on the concentration-dependent viscosity [35] of γ-PGA, a high amount of γ-PGA was applied to reduce the inhomogeneous dispersion of HA in printing. Obviously, all the loss moduli of hydrogels were smaller than the storage modulus (G’’ < G’), which is a suitable gel character for 3D direct-write extrusion printing [36] (Fig. 3A).

The fundamental characteristic of a 3D scaffold is mechanical properties, our materials possess a significantly higher young modulus compared to Wong et al.'s (2017) report with the young modulus of the articular collagen type II construct was 9.86 kPa [37]. The Young’s modulus of a scaffold is very important for bone tissue engineering because of its simultaneous reconstruction and load-bearing function at the same time. The Col-HA group showed the increasing load-bearing character owing to the addition of HA in this composite and the alignment of materials in 3D shape with high exposure in the cross-linker due to their porosity. Although the Col-HA-M membrane has HA in its structure, the compressive modulus is still low because of the 2D structure, which lowers the density of the cross-linker making it a better load-bearing candidate. Even compared to the Col and Col-P scaffolds, the compression stress of the 2D membrane is lower, this demonstrates the 3D scaffolds’ porosity is a central factor that induces their mechanical properties.

Initially of biocompatibility testing, after immersion in SBF, apatite was formed in the Col, Col-P, and Col-HA scaffolds as well as the Col-HA-M membrane. However, the Col-HA-M scaffold showed a greatly reduced amount of apatite after 14 and 21 days of immersion as the intensity peak decreased (Fig. 5) due to decomposition. This is the consequence of less cross-linking between the high density of polymer compounds in the 2D membrane. Obviously, a lower molecular density leads to higher cross-linker formation in the 3D scaffolds. Otherwise, the apatite layer supports osteogenesis owing the formation of a tight chemical bond between osteocytes and materials [26]. Thus, the 3D scaffolds present a better possibility of biocompatibility in general.

As the low mechanical properties of collagen, the OPC crosslinker was introduced into the system to maintain the mechanical stability of the biomaterials. If the degradation rate is too high, there is insufficient mechanical strength to support the osteocyte/chondrocyte formation. However, if the degradation is too low it cannot create sufficient space for cell growth. Our biocompatible materials are stronger than the CS/HAp reported by Nguyen et al*.* group, which decomposed up to 46.37% after 28 days of immersion in PBS [38]. The degradation rates of Col-HA and Col-HA-M were higher because of the greater released rate of hydroxyapatite. This can be explained by the interaction between collagen and hydroxyapatite is the self-organization of the hydroxyapatite directional deposition on collagen and surface interaction in the composite. The direction between HAp and collagen is restricted by the covalent bond between COO^−^ and Ca^2+^ to maintain regular coordination [39]. Once this bonding is disturbed, the hydroxyapatite will be released. This is completely different from the multi-bonding between collagen and γ-PGA with polyelectrolyte complexes that consist of collagen as a polyelectrolyte cationic and γ-PGA as the polyelectrolyte anionic [25]; Multiple hydrogen bonds are formed between OPC (-OH) and Collagen (-HO and -N2H) [40].

Then the cell viability was influenced by the mechanical and chemical characteristics of the scaffolds. This could be explained by hydroxyapatite of Col-HA enhancing the mechanical properties of the scaffold, which can induce significant growth of the cell lines [41]. Although the Col-HA-M membrane group showed good cell viability, the Col-HA group presented the greater cell proliferation since the ideal macropore size of the 3D scaffold plays the role mimicked the microenvironment for both rat mesenchymal stem cells and human adipose-derived stem cells growth. Osteogenesis and chondrogenesis of Col-P and Col-HA were evaluated by gene expression for further information. As mentioned by Yang et al*.* (2020), the Ca^2+^ ions released from hydroxyapatite expedite osteoconductive and osteoinductive processes via FAK-ERK pathway activation [5]. In addition, Ca^2+^ and L-arginine work together to express the coupling pathway of NO/cGMP during osteogenesis and angiogenesis [42, 43]. BMP-2 is a potent osteogenic factor that promotes the differentiation of mesenchymal stem cells into fibroblasts and chondroblasts [44] Moreover, BMP-2 and BMP-4 divert aortic calcifying vascular cells to osteogenic fates [45] [46]. The amount of BMP-2 specifies the characteristics of materials in the differentiation stem cells for both initial and later phases, especially in the Col-HA group.

In animal trials, we used the rat model to mimic the clinical microenvironment that provides humidity, relevant nutrients, gaseous concentrations, and growth factors [47] for cytotoxicity testing. While the nude mice's lack of thymus results in T-cell deficiency and immunodeficient, thereby being able to accept foreign tissue [48, 49] and the formation of blood vessels appears from the second week. This demonstrates that our materials are highly biocompatible with both the healthy and immunodeficient individuals. Even though the ectopic subcutaneous implant is the least invasive model, it can be used for primary screening of MSCs from different tissue sources and new scaffold materials [49, 50]. In addition, blood vessel formation greatly supports spontaneous regeneration. There is potential healthy crosstalk between the subchondral bone and the articular cartilage, which leads to coupled bone remodeling in order to preserve homeostasis and repair microdamage [51].

Although the Col-HA-ℽ-PGA scaffold demonstrated excellent mechanical properties, biocompatibility, and upregulation of osteochondrogenesis, the specific mechanism by which they affect the immune system and metabolism as well as the cell morphology remain elusive. Otherwise, the mechanical properties of the scaffold were improved by addition of hydroxyapatite in the 3D structure, which still must be enhanced by other cross-linker to obtain a higher Young’s modulus similar to the Young’s modulus of the native bone. Furthermore, we will focus on site-specific implants in our future studies to gain a better understanding of the load-bearing and biocompatible characteristics associated with the in-situ regeneration of bone and osteochondral tissues.

## Conclusion

This study focused on the preparation of a Col/HAp/ℽ-PGA composite using a 3D printing technique to fabricate 8 mm x 8 mm × 1.2 mm multilayer scaffolds. It is possible to print a multilayer scaffold with the first layer consisting of HA and ℽ-PGA mixture, and the 2nd to 4th layers consisting of collagen-ℽ-PGA solution using two printheads of a 3D direct-write bio-printer. The scaffold exhibited good biodegradation with a weight loss of up to 26%. Owing to the 3D structure and OPC crosslinker, the mechanical properties of the Col-HA scaffold were increased five times better than those of the 2D membrane. The biomaterial scaffold and membrane showed good biocompatibility in FE-SEM and XRD when immersed in the stimulated body fluid, along with the CCK-8 assay. The results of genes-specific differentiation showed that the Col, Col-HA scaffolds stimulated *r*BMSC for bone regeneration, in contrast, Col-P improved the growth of the cells for cartilage restoration. The Col-HA possesses the potential of osteogenic via stimulated BMP-2 expression and encouraged *h*ADSCs proliferation and differentiation. Thus, this study developed a feasible way to fabricate the multilayer scaffold that combines ceramic and polymer matrix for bone regeneration and potential of osteochondral regeneration.

## Data Availability

The datasets used and/or analyzed during the current study are available from the corresponding authors on reasonable request.

## Abbreviations

γ-PGA: γ-Polyglutamate acid
3D: Three dimensions
2D: Two dimensions
*r*BMSCs: Rat bone mesenchymal stem cells
*h*ADSCs: Human adipose-derived stem cells
OCD: Osteochondral defect
HA: Hydroxyapatite
OPCs: Oligo Proanthocyanidins
FT-IR: Fourier-transform infrared spectroscopy
XRD: X-Ray Diffraction
FE-SEM: Field Emission Scanning electron microscope
MCR: Modular Compact Rheometer
PBS: Phosphate Buffer Saline
FBS: Fetal Bovine Serum
SBF: Simulated Body Fluid
SD: Sprague Dawley
DMEM: Dulbecco’s Modified Eagle’s Medium
BMP-2: Bone morphogenetic protein 2
IACUC: Affiliated Institutional Animal Care and Use Committee
H&E: Hemoxylin and Eosin
CCK-8: Cell counting kit-8
